# Learning effects during balance analysis on a modified posturomed-platform in healthy dogs

**DOI:** 10.1186/s12917-025-05257-y

**Published:** 2026-01-08

**Authors:** Wolszky Viola, Zablotski Yury, Lauer Susanne

**Affiliations:** 1 Small Animal Department, Faculty of Veterinary Medicine, Leipzig University, Leipzig, 04103 Germany; 2 LMU Small Animal Clinic, Centre for Clinical Veterinary Medicine, Ludwig-Maximilians-University Munich, Munich, 80539 Germany

**Keywords:** Posturography, Dogs, Posturomed, Learning effects, Center of pressure

## Abstract

**Background:**

Posturographic balance assessments are increasingly used in veterinary medicine, yet potential learning effects during evaluation remain unstudied. This study investigated learning effects in static, slow-dynamic, and fast-dynamic posturography using a modified Posturomed platform in healthy dogs.

**Material and methods:**

Healthy adult dogs (*n* = 20) were positioned longitudinally on a pressure sensitive modified balance platform (Posturomed-FDM-JS, Zebris, Isny, Germany). Five static, slow-dynamic and fast-dynamic posturographic trials were recorded (duration: 20s) and repeated three times over three weeks. Center of pressure (COP) parameters—COP-path-length (PL, mm), 95% COP-confidence-ellipse-area (CEA, mm^2^) and COP-average-velocity (AV, mm/sec) were analyzed over five steady-state 5-s intervals per trial. Data were analyzed using generalised linear or robust linear mixed-effects models with random effects on the individual dog; p-values were adjusted using the Tukey method for multiple comparisons.

**Results:**

Under static conditions, none of the COP-parameters differed significantly across time points (all *p*-values > 0.448). Under slow-dynamic conditions, all COP-parameters decreased significantly between time points 1 and 2 (all *p* values < 0.0007) but remained stable thereafter (all *p*-values > 0.159). Under fast-dynamic conditions, all COP-parameters decreased significantly from time point to time point (all *p*-values < 0.034), except for CEA between time points 1 and 3 (*p* = 0.0759) and 2 and 3 (*p* = 0.999). Differences among trials occurred only at the first time point under dynamic conditions and were more pronounced under fast-dynamic conditions.

**Conclusions:**

No learning effects were observed during static posturography in healthy adult dogs. However, training effects must be considered in both slow- and fast-dynamic posturography.

**Supplementary Information:**

The online version contains supplementary material available at 10.1186/s12917-025-05257-y.

## Background

Balance evaluation has become a cornerstone in veterinary medicine for detecting changes in body or limb strength, stability, coordination, and equilibrium in response to various orthopedic or neurological diseases, as well as age-related postural deficits [1–8]. Balance can be defined as the ability to maintain or restore the body’s center of gravity within the base of support through coordinated sensory and motor responses. Static balance refers to maintaining postural stability during quiet standing on a stable surface, whereas dynamic balance involves controlling posture during movement or external perturbations. Both depend on limb strength, joint stability, proprioceptive input, and neuromuscular coordination, which together determine postural equilibrium and stability [9–11]. This assessment plays a crucial role in evaluating rehabilitation outcomes, identifying balance deficits associated with specific conditions, and implementing targeted interventions to address them [1, 2].

Historically, balance evaluation in dogs has been predominantly subjective [1, 2]. Over the past two decades, the introduction of static and dynamic posturography in veterinary medicine has enabled objective and quantitative evaluation of balance performance [3, 12–16]. Posturography measures fluctuations of the center of pressure (COP), which represents the centroid of the vertical ground reaction forces projected onto the horizontal plane [17, 18]. The COP serves as a dynamic indicator of postural control, reflecting the coordinated activity of the musculoskeletal and neuromuscular systems that maintain equilibrium [19]. In animals, posturographic balance assessments obtained through stance and gait analyses provide reliable measurements, both on static platforms and instrumented treadmills [3, 12–15, 20, 21]. Static posturography quantifies postural control by recording COP displacements in upright stance quiet on a stable surface [22, 23], whereas dynamic posturography evaluates the body’s ability to maintain stability in response to controlled perturbations. Analysis of COP trajectories allows detection of subtle balance impairments or weight distribution changes associated with aging, sensory deficits, or orthopedic conditions [3–7]. Recently, static posturographic measurements in dogs standing quietly on a flat, pressure sensitive platform demonstrated excellent inter-observer reliability across three trials conducted in a single day [15]. Static posturographic measurements using the Wii Balance Board (Nintendo, Japan) have been validated for dogs, and measurements obtained on the modified Posturomed platform (Zebris, Isny, Germany) have been shown to be reliable in healthy dogs [24–26]. However, because bipedal stance on a solid surface is relatively stable and not enough challenging, static balance tests are limited in their ability to detect functionally relevant improvements in postural control and small differences in balance ability in humans [27, 28]. When a more comprehensive assessment of postural control is needed, dynamic posturography is preferable to static posturography, as it can detect subtle impairments and assess the adaptability of postural control mechanisms by introducing balance challenges through perturbations [28–30].

In dynamic posturography, balance is typically challenged by inducing sudden lateral or rotatory perturbations using instrumented computerized movable support platforms or treadmill systems, thereby testing and challenging the anticipatory and compensatory balance mechanisms [16, 25, 31]. In veterinary medicine, dynamic posturography is increasingly used in dogs for both research and clinical rehabilitation. This includes assessments performed while dogs walk on instrumented platforms or treadmills or stand on platforms equipped with perturbation mechanisms such as the Imoove-vet® and Posturomed platforms [13, 14, 16, 25, 32]. Currently, dynamic balance assessment and training in dogs primarily rely on treadmills without external perturbation mechanisms [13, 14, 32]. However, pressure-sensitive treadmills with adjustable swing crossbeams for perturbation are now being introduced in veterinary sports medicine and physiotherapy, though their effects have yet to be studied [33, 34]. Traditional treadmills challenge balance by requiring continuous postural adjustments to maintain a steady gait, altering sensory feedback, and inducing slight gait variations that engage the neuromuscular system [35–37]. In contrast, perturbation-capable treadmills introduce sudden changes in belt speed, platform shifts, or oscillations, disrupting gait stability and demanding adaptive responses [37, 38]. Humans adjust to these perturbations using feedback and feedforward strategies, primarily by modifying foot placement and center of mass control [38, 39].

The Imoove platform (Allcare Innovations, 26,500 Bourg les Valence, France) is a multi-axis posturographic system for use in dogs, allowing for dynamic balance assessment and training using various perturbations, including tilt, rotation, and translation. While the Imoove has been shown to challenge postural stability in healthy dogs, with increased platform amplitude impacting COP parameters more significantly than speed alterations, the reliability of this system has not been assessed yet for balance assessment in dogs [16]. Compared to the Imoove system, the modified Posturomed platform only allows for regular monodirectional horizontal perturbations. Training on the Posturomed has been shown to improve muscle coordination and stability during stance, underscoring its role in enhancing neuromuscular control in human medicine [40, 41]. In healthy dogs, dynamic posturographic assessments using the modified Posturomed platform revealed a significant decrease in COP parameters between two consecutive time points, suggesting a training or learning effect similar to that observed in humans [25, 42, 43].

Repeated balance assessments in humans frequently result in improved performance that does not necessarily reflect genuine enhancements in balance capacity, but rather short-term procedural habituation to the testing setup [43–46]. This habituation effect represents a temporary behavioral adjustment to repeated exposure rather than true motor learning, which involves lasting improvements in postural control through practice or training. In the context of this study, the term ‘learning effect’ refers to short-term habituation to repeated testing, whereas ‘training effect’ denotes longer-term improvement in postural control through practice or conditioning. Balance training in healthy adults improves balance ability irrespective of task difficulty, supported by evidence that both low- and high-difficulty training conditions produce significant performance gains with no statistically significant differences between groups [47].

However, improvements in postural stability are not linear and are strongly influenced by the characteristics of the specific balance task. As task complexity increases, corresponding changes occur in postural sway and neuromuscular responses in a non-linear manner, indicating that the mechanisms of motor adaptation are task-dependent [48]. In this context, the so-called *"learning to learn"* effect—where prior training facilitates the acquisition of new but related motor tasks—remains controversial in balance training. Prior exposure to balance tasks does not reliably accelerate learning of novel balance challenges. A single practice session is typically insufficient to elicit meaningful improvements in balance ability. Instead, individual physical factors, particularly lower limb power, are more predictive of both learning rates and overall balance performance across repeated trials [49].

In summary, balance performance in healthy adults improves with repeated practice across different task conditions. These improvements unfold in a non-linear, task-specific manner, and current evidence does not robustly support a generalized *"learning to learn"* effect in balance training. Rather, individual motor attributes such as lower limb strength appear to be more reliable predictors of performance gains and learning dynamics [47–49].

In dynamic posturography, habituation effects, especially between the first and second trials, are well-documented in human medicine. As a result, initial trials are often excluded or dedicated acclimatization phases are implemented to reduce test habituation effects [42, 44]. These habituation phenomena are typically characterized by increased center-of-mass displacement and elevated electromyographic activity during initial assessments [44]. In contrast to humans, dogs adopt a quadrupedal stance that distributes body weight over four limbs, resulting in different postural control strategies and center-of-pressure dynamics. These biomechanical differences limit the direct transferability of findings from bipedal to quadrupedal balance research. Studies using the Posturomed platform in humans have demonstrated significant intra-day variability but consistent inter-day results, indicating that most habituation occurs during the first measurement session rather than between days [43]. Nonetheless, in people, balance skills can be acquired relatively quickly. For instance, healthy individuals typically adapt to standing on a multiaxial balance board within two sessions and retain this ability after one week [46]. Similarly, patients with Parkinson’s disease exhibit progressive improvements over successive balance assessments, with performance gains continuing beyond the fourth session before reaching a plateau [50]. Emotional factors such as anxiety and arousal may also influence initial balance performance, but their effects tend to diminish with repeated exposure, potentially contributing to perceived improvements across trials [51].

Since a learning effect can lead to an underestimation of true balance ability in initial assessments and an overestimation in later ones, it is important to determine its impact on balance testing in dogs. To the authors’ knowledge, the progression of learning effects beyond two assessment points in dynamic balance analysis remains unstudied in veterinary medicine. This study aimed to determine whether learning effects persist beyond the second assessment and to evaluate the impact of perturbation frequency. We hypothesized that a modified Posturomed balance platform allows for balance assessments in healthy adult dogs without additional learning effect beyond the third assessment.

## Results

### Dogs

The study sample consisted of seven mixed breed dogs, two longhaired Whippets and one of each of the following breeds: Beagle, FBI, Labrador-Mix, Shepherd-Mix, Tibet Terrier, Lagotto Romagnolo, Weimaraner, Goldendoodle, Rottweiler, Miniature Australian Shepherd, Waller. Six dogs were males and 14 were females of which eight were spayed females, four were neutered males, six dogs were intact females and two were sexually intact males. The dogs had a mean age of 5.5 ± 2.8 years. Mean shoulder height was 51.3 ± 8.1 cm. Mean body mass was 19.7 ± 6.9 kg. All dogs had a body conditioning score between 4/9 and 6/9 [52]. The mean interval between the first and second time points was 1.3 ± 0.6 days, between the second and third was 6.7 ± 2.8 days, and between the third and fourth was 1.2 ± 0.4 days. The overall mean duration covering all four time points was 9.1 ± 2.8 days. All dogs successfully completed five valid trials, and none were excluded from the study.

### Acclimatization and trial duration

The mean acclimatization times were 2.8 ± 0.7 min at the first time point, 1.7 ± 0.7 min at the second, 1.5 ± 0.6 min at the third, and 1.8 ± 0.9 min at the fourth. The mean duration of the posturographic measurements was 24.8 ± 6.7 min at time point one, 20.1 ± 8.0 min at time point two, 21.0 ± 8.5 min at time point three, and 19.5 ± 11.7 min at time point four.

### Changes in COP parameters across the four time points

Under static conditions, there were no significant differences in any of the three COP parameters (CEA, PL, AV) across the four time points (*p* ≥ 0.448, Tables 1 and 2). Under slow-dynamic conditions, all three COP parameters decreased significantly between time points 1 and 2 (*p* ≤ 0.0007), but no significant differences were observed between time point 2, 3 and 4 (*p* ≥ 0.1596). Under fast-dynamic conditions, all COP parameters decreased significantly between time points 1 and 2 (*p* ≤ 0.0336). The CEA did not differ significantly between time points 1 and 2 (*p* = 0.1104) and 2 and 3 (*p* = 0.9993) but decreased significantly from time point 3 to time point 4 (*p* = 0.0033). The other two COP-parameters, PL and AV, decreased significantly between time points 2 and 3 and time point 3 and 4 (*p* ≤ 0.0099).

**Table 1:** Center of pressure (COP) parameters across four time points: Mean values with lower and upper limits of the 95% Confidence Interval [95% CI] for the COP confidence ellipse area (CEA), path length (PL) and average velocity (AV) under static, slow dynamic and fast dynamic conditions

Condition	COP parameter	Time point 1 Mean [95% CI]	Time point 2 Mean [95% CI]	Time point 3 Mean [95% CI]	Time point 4 Mean [95% CI]
Static	CEA (mm^2^)	303 [98.7–507]	303 [94.6–512]	307 [101.3–512]	310 [105.8–515]
	PL (mm)	455 [398–513]	432 [375–489]	432 [375–489]	429 [372–486]
	AV (mm/sec)	91.1 [79.7–102.5]	86.4 [75–97.8]	86.3 [74.9–97.7]	85.7 [74.3–97]
Slow-dynamic	CEA (mm^2^)	1913 [1656–2170]	1444 [1212–1675]	1451 [1175.8–1727]	1291 [1059.3–1522]
	PL (mm)	895 [838–952]	768 [711–825]	740 [683–797]	730 [673–788]
	AV (mm/sec)	178.6 [167.2–190]	153.6 [142.2–164.9]	147.9 [136.5–159.3]	146 [134.6–157.3]
Fast-dynamic	CEA (mm^2^)	3038 [2624.2–3452]	2408 [2067.9–2748]	2400 [2081–2720]	2004 [1720.7–2288]
	PL (mm)	1222 [1164–1279]	1089 [1032–1146]	1031 [974–1088]	974 [917–1031]
	AV (mm/sec)	244.1 [232.7–255.5]	217.6 [206.2–229]	206 [194.6–217.4]	194.5 [183.1–205.9]

**Table 2:** Comparison of center of pressure (COP) parameters across four time points: COP confidence ellipse area (CEA), path length (PL) and average velocity (AV) under static, slow dynamic and fast dynamic conditions. *P*-values < 0.05 are considered as significant

Condition	COP parameter	Comparison between time points Estimates [CI 95%] p values					
		1.−2	2.−3	3.−4	1.−3	1.−4	2.−4
Static	CEA (mm^2^)	−4.72 [−52.8–43-3] 0.9943	−6.84 [−30.9–17.3] 0.8850	−1.93 [−39.4–35.6] 0.9992	−11.56 [−62.9–39.8] 0.9382	−13.49 [−62.4–35.5] 0.8935	−8.77 [−42.5–24-9] 0.9086
	PL (mm)	23.05 [−24.04–70.1] 0.5891	0.33 [−46.76–47.4] 1.0000	3.28 [−43.76–50.4] 0.9979	23.38 −23.71–70.5] 0.5777	26.7 [−20.38–73.8] 0.4629	3.65 [−43.43–50.7] 0.9972
	AV (mm/sec)	4.68 [−4.72–14.07] 0.5755	0.08 [−9.31–9.47] 1.0000	0.66 [−8.73–10.05] 0.9979	4.75 [−4.62–14.14] 0.5616	5.41 [−3.98–14.80] 0.4481	0.74 [−8.65–10.13] 0.9971
Slow-dynamic	CEA (mm^2^)	506.35 [169.3–843.4] 0.0007	15.44 [−262.0–292.9] 0.9990	133.8 [−31.5–299.2] 0.1596	521.8 [159.9–883.7] 0.0012	655.6 [317.6–993.6] <.0001	149.25 [−99.8–398.3] 0.4127
	PL (mm)	126.63 [79.54–173.7] <.0001	28.33 [−18.76–75.4] 0.4092	9.59 [−37.49–56.7] 0.9533	154.96 [107.87–202.0] <.0001	164.55 [117.47–211.6] <.0001	37.92 [−9.16–85.0] 0.1629
	AV (mm/sec)	25.01 [15.62–34.40] <.0001	5.68 [−3.71–15.07] 0.4043	1.92 [−7.48–11.31] 0.9531	30.69 [21.30–40.08] <.0001	32.6 [23.21–41.99] <.0001	7.6 [−1.80–16.99] 0.1599
Fast-dynamic	CEA (mm^2^)	647.3 [−92.5–1387.1] 0.1104	15.82 [−302.2–333.9] 0.9993	341.68 [86.4–596.9] 0.0033	663.12 [−42.9–1369.2] 0.0746	1004.79 [390.8–1618.8] 0.0002	357.49 [19.2–695.8] 0.0336
	PL (mm)	132.73 [85.64–179.8] <.0001	58.01 [10.92–105.1] 0.0085	57.13 [10.05–104.2] 0.0099	190.73 [143.65–237.8] <.0001	247.87 [200.78–295.0] <.0001	115.14 [68.06–162.2] <.0001
	AV (mm/sec)	26.46 [17.07–35.85] <.0001	11.63 [2.24–21.02] 0.0080	11.45 [2.06–20.84] 0.0095	38.1 [28.70–47.49] <.0001	49.54 [40.15–58.93] <.0001	23.08 [13.69–32.47] <.0001

### COP parameters – time point-specific intertrial variability

**Fig. 1:**
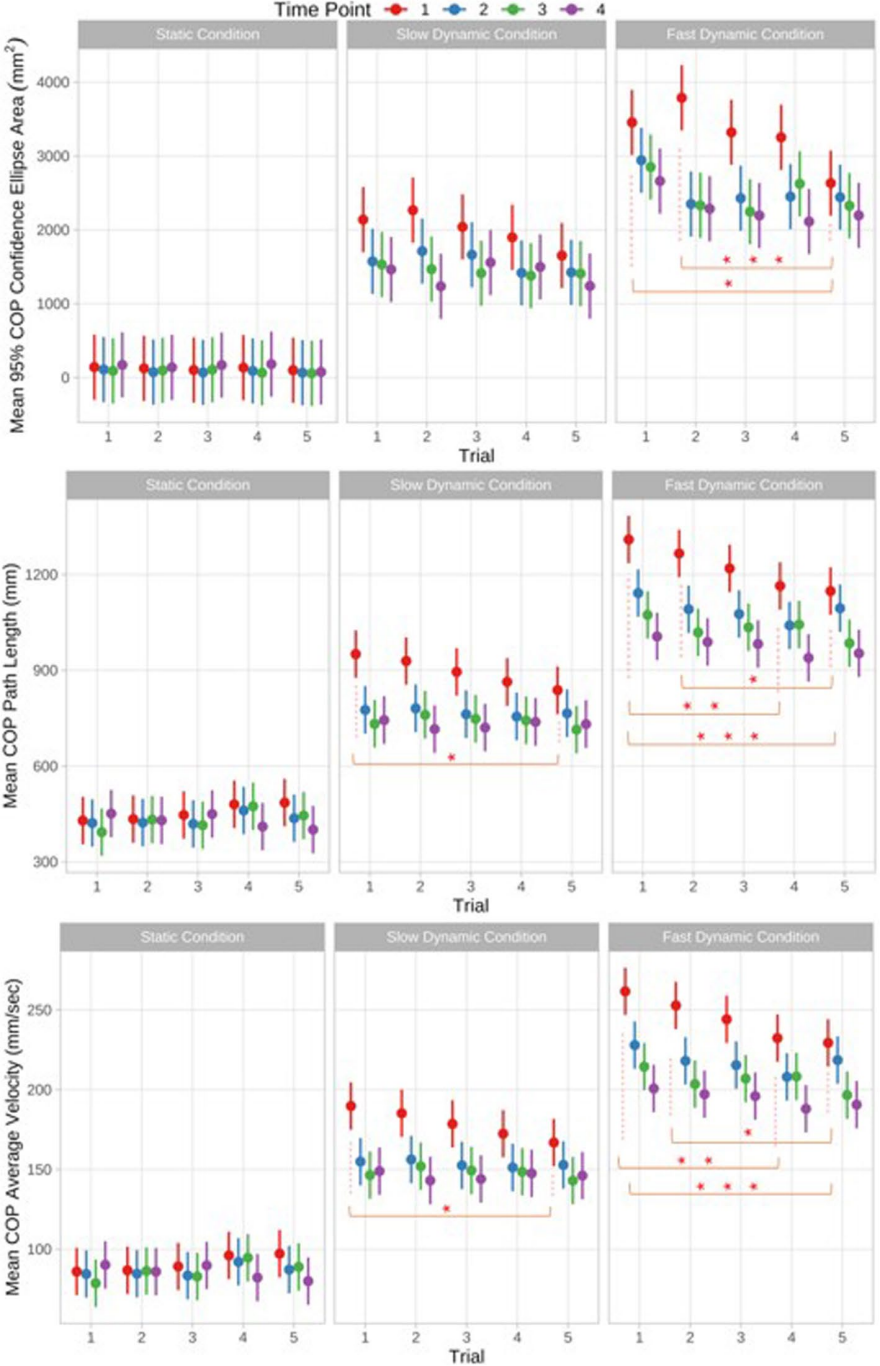
Center of pressure (COP) parameters: Time point-specific intertrial variability for COP confidence ellipse area, path length and average velocity across four time points under static, slow dynamic and fast dynamic conditions. Estimated marginal means of mixed- and robust-mixed models are displayed with 95% CI. Tukey correction was used. Significant differences are indicated by asterisks: * for *p* < 0.05, ** for *p* < 0.01, and *** for *p* < 0.001

Under static and slow dynamic conditions, the 95% COP confidence ellipse (CEA) did not differ significantly between trials at any time point (Fig. 1). Under fast dynamic conditions, at the first time point, CEA was significantly lower in trial 5 compared to trial 1 (*p* = 0.0368) and to trial 2 (*p* = 0.0007). but showed no significant differences among the other trials. At all subsequent time points, no further significant differences between trials were observed.

Under static conditions, path length (PL) did not differ significantly between trials at any time point. Under slow dynamic conditions, at the first time point, PL was significantly shorter in trial 5 compared to trial 1 (*p* = 0.0442) but showed no significant differences among the other trials. At all subsequent time points, no further significant differences between trials were observed.

Under fast dynamic conditions, at the first time point, PL was significantly longer in trial 1 compared to trial 4 (*p* = 0.0034) and trial 5 (*p* = 0.0007), and significantly longer in trial 2 compared to trial 5 (*p* = 0.0314). No significant differences were observed among the other trials. At all subsequent time points, no further significant differences between trials were observed.

Under static conditions, average velocity (AV) did not differ significantly between trials at any time point. Under slow dynamic conditions, at the first time point, AV was significantly slower in trial 5 compared to trial 1 (*p* = 0.0387) but showed no significant differences among the other trials.

Under fast dynamic conditions, at the first time point, AV was significantly faster in trial 1 compared to trial 4 (*p* = 0.0029) and trial 5 (*p* = 0.0007), and significantly faster in trial 2 compared to trial 5 (*p* = 0.0324). No significant differences were observed among the other trials. At all subsequent time points, no further significant differences between trials were observed.

## Discussion

Objective evaluation of balance ability and its progression after training or rehabilitation in dogs requires understanding of potential learning effects from repeated balance assessments. Due to the limited scientific evidence on learning phenomena in canine balance testing and posturography, this study examined how repeated assessments influence posturographic measurements under static and dynamic conditions across four time points. The results supported our initial hypothesis for both static and slow dynamic conditions. In static posturography, no significant learning effects were observed across repeated assessments. Under slow dynamic conditions, the COP parameters – confidence ellipse area (CEA), path length (PL) and average velocity (AV) – did not differ between the second, third and fourth time points, but all decreased significantly between the first and second time point, indicating an initial familiarization effect. More strikingly, under fast dynamic conditions, all COP parameters decreased significantly across successive time points except for CEA between time points 1 and 2 and 2 and 3.

The static findings align with earlier reports demonstrating that static posturographic measurements and stance analyses can be reliably assessed in dogs and horses [3, 5, 7, 20]. Unlike these studies, the unlocked Posturomed allows for undulating movements even during static assessments due to its horizontally free-swinging nature [25]. The lack of significant differences in COP parameters across static trials suggests that a single measurement is sufficient once adequate acclimatization has been achieved. Acclimatization and total testing times were longest at the first time point and declined thereafter, indicating rapid habituation and stable posturographic behavior for static assessments. The longer durations during the first session likely reflected behavioral familiarization rather than inconsistent testing conditions, as all procedures were standardized and supervised by the same operator. Static posturography inherently provides less postural challenge than dynamic assessments, but this stability can be advantageous for evaluating dogs with reduced balance capacity, such as those with neurological disorders, because it enables assessment without excessive physical demand. Conversely, static testing may not be sufficiently challenging to detect subtle balance differences or long-term training adaptations in healthy or athletic dogs, for which dynamic or perturbation-based assessments are more appropriate.

In contrast to a previous canine study conducted on completely static platforms [15], which required multiple short trials to achieve reliable results depending on the parameter and recording duration, our findings may be explained by methodological differences. This earlier study analyzed both limb and body parameters, whereas the present investigation focused solely on global body COP parameters, which are likely less affected by localized stance variations and therefore may show lower measurement variability [15].

This study intentionally focused on global COP parameters (CEA, PL, and AV), as the modified Posturomed platform applies monodirectional lateral deflections that could bias directional COP measures and reduce interpretability. Evaluating global COP parameters therefore provided a direction-independent and comprehensive measure of postural stability, methodologically consistent with previous Posturomed research.

Both dynamic conditions revealed significant inter-trial differences only at the first time point, with effects more pronounced under fast-dynamic conditions. This indicates that dogs require a familiarization period before producing reliable results in dynamic posturography on the modified Posturomed platform. At higher perturbation frequencies, as used under fast-dynamic conditions in the present study, dogs may require a longer familiarization period to achieve stable performance across repeated evaluations. Under static conditions, posturographic parameters were stable from the first evaluation, confirming that repeated static measurements are not necessary once initial acclimatization has occurred. Acclimatization times were longest during the first dynamic evaluation and decreased thereafter, reflecting individual variation in adaptability and confirming that a preparatory session is beneficial before data collection under dynamic conditions. These findings demonstrate that the modified Posturomed platform is well tolerated by dogs of different breeds and allows reliable static and dynamic posturographic assessments after brief acclimatization. The observed variability among dogs emphasizes the importance of standardized acclimatization procedures and the inclusion of a familiarization phase in future protocols. While this study confirmed the reliability of the Posturomed platform for short-term repeated assessments, formal validation across larger and clinically affected populations will be required to confirm its diagnostic applicability.

Prior studies using stable treadmills or locked pressure-sensitive mats confirmed reliable dynamic posturographic measurements in both healthy and lame dogs while walking on these surfaces [13, 14, 32]. More recently, dynamic balance responses were evaluated in healthy dogs subjected to combined rotational, eccentric and inclinational movements on a motorized platform (Imoove-vet® platform, Allcare Innovations, Bourg les Valence, France). The authors reported that pertubations induced by the Imoove-vet® significantly affected COP parameters suggesting the platform posed substantial challenges in maintaining posture [16]. In contrast, dogs in our study adapted well to both dynamic conditions on the modified Posturomed platform, consistent with findings from a previous study using comparable equipment [25]. These findings suggest that the modified Posturomed platform is well tolerated across breeds and allows consistent posturographic assessments after brief acclimatization, supporting its feasibility for static and dynamic balance evaluation in healthy dogs. A recent study evaluated postural stability in healthy dogs using a four-week proprioceptive training program on a motorized platform generating three-dimensional elliptical perturbations. The authors reported significant reductions in several COP parameters in the training group, particularly under challenging conditions, while the control group showed only minor changes. These findings confirm the effectiveness of repeated proprioceptive training in improving postural stability and differ from the present study mainly in their long-term interventional design compared with the short-term adaptive assessments performed here [53].

In our study, learning effects were particularly pronounced between the first and the second time point during both dynamic conditions, consistent with human studies showing significant adaptation after the initial exposure [42, 43]. In people, initial trials in posturography often show increased sway amplitudes and greater variability, which decrease with repeated testing reflecting short term habituation effects [42]. Thus, in most human dynamic posturography studies, the initial trial is typically excluded to mitigate habituation effects, which are especially pronounced during the first trial [44]. Previous studies have emphasized the importance of allowing sufficient time for acclimatization to the posturographic device before data collection [42, 44]. In the present study, dogs were given individualized acclimatization periods based on the owners’ judgment and performed a test trial prior to data recording. Acclimatization times decreased across successive sessions, likely reflecting increasing familiarity with the testing environment and procedures. These differences were not analyzed statistically, as the study focused on the progression of COP parameters. Our protocol differed from human studies by using continuous rather than sudden perturbations, which enhanced canine acceptance but may have allowed some habituation during repeated trials [25].

Pre-exercise balance training can also induce task-specific motor adaptations, facilitating motor learning during the assessment [45]. This phenomenon has not been systematically studied in dogs yet. In people, electromyographic amplitudes decrease after approximately 15 s of balance exercises, leading to recommendations to limit individual trials to this duration [41]. In our study, each trial lasted 20 s, which may be a limitation. However, the onset of fatigue in dogs has not been systematically studied yet. Although this duration was well tolerated based on preliminary trials [25], another recent study demonstrated that one 10-s or two 5-s recordings can yield reliable results in static posturography [15].

Based on our results, the Posturomed platform is suitable for balance assessment and for monitoring in individual healthy dogs under both static and slow-dynamic conditions. While static assessments yield reliable results from the first trial, a preparatory trial is advisable under slow-dynamic conditions to ensure measurement reliability. Under fast-dynamic conditions, performance gains extended across all four time points, indicating continued adaptation. These findings suggest that higher perturbation frequencies place greater demands on postural control and may more effectively promote balance training and muscle strengthening. This interpretation is supported by previous studies in humans, where dynamic posturography on the Posturomed increased muscle activity, particularly when stability was further challenged through single-leg stance [41]. Similarly, structured training on the Imoove-vet® platform led to limb circumference increases in agility dogs, suggesting muscle mass gains and reinforcing the idea that training effects may result from muscular improvement [54]. Unlike our short-duration study, that protocol involved bi-weekly training for six weeks. Hence, our design did not allow conclusions regarding long-term muscular effects. Consistent with these findings, a more recent study demonstrated that proprioceptive training on the Imoove-vet® platform reduces COP excursions, indicating improved postural stability [53]. Future studies should investigate whether similar musculoskeletal adaptations can be achieved using the Posturomed over prolonged training periods.

This study was limited to healthy dogs. Further research is needed to evaluate the response of dogs with orthopedic or neurological impairments to posturographic assessments on the Posturomed platform. Although healthy dogs show effective balance adaptation under perturbations [16, 25], it remains unclear how disease conditions might affect this capacity.

The mechanisms underlying the observed learning effect may involve habituation and improved neuromuscular or sensory integration, as seen in humans [41, 42, 44]. For instance, a recent human study showed that balance training enhances the ability to perform comparable motor tasks, likely due to improved integration of somatosensory and vestibular inputs [45]. Overall, the vestibular system appears to play a central role in both initial trial effects and ongoing stabilization during repeated perturbations [44, 55]. Interestingly, muscles with greater vestibular sensitivity tend to exhibit more rapid adaptation [44].

While unfiltered COP data may include higher-frequency components, the proprietary distance-threshold algorithm (“Russian hops”) implemented in the WinFDM 1.2.2 software partially mitigated sensor noise. Recent evidence indicates that applying a 6 Hz low-pass Butterworth filter yields optimal data quality for standing dogs [56]. Future studies using newer software versions with adjustable filters should adopt such standardized processing to enhance comparability and reduce noise-related variability.

Finally, several additional limitations must be acknowledged. The heterogeneity in breed, conformation, and physical fitness of the participating dogs may have influenced COP parameters and learning effects. In human medicine, fitness levels can affect posturographic measurements [42], and in veterinary medicine, age and health status are already known factors influencing balance ability [3, 57, 58]. In addition, minor procedural variation occurred during dynamic trials, as some dogs were gradually accustomed to increasing oscillation frequency while standing on the platform, whereas others stepped onto the platform after the target frequency had been reached. This variation was based on individual comfort levels and may have introduced slight differences in initial balance performance, as dogs more confident in stepping directly onto the moving platform could have demonstrated superior baseline stability. However, all dogs adapted rapidly during the testing sessions, and this variability is unlikely to have substantially affected the overall results. Further research is needed to evaluate these potential effects systematically and to determine the long-term impact of Posturomed training in dogs, particularly in those with neurological or orthopedic conditions. These findings collectively clarify the short-term postural adaptations that occur in healthy dogs during repeated static and dynamic balance assessments.

## Conclusion

Posturographic measurements obtained under static conditions using the modified Posturomed balance platform in healthy dogs provided reliable results from the first trial, with minimal to no effects. Under slow-dynamic conditions, the platform allowed for consistent measurements, as indicated by the absence of significant differences beyond the second assessment. In contrast, under fast-dynamic conditions, learning effects were evident across repeated assessments for most COP parameters, suggesting ongoing adaptation over time.

Overall, the modified Posturomed balance platform proved feasible and reliable for static and slow-dynamic posturographic assessments in healthy dogs. These findings indicate its potential for objective balance evaluation and for use as both a diagnostic and training tool in veterinary rehabilitation. Future research may benefit from applying optimized filtering approaches to refine comparability of COP parameters across studies and to validate the platform’s application in dogs with orthopedic or neurologic conditions.

## Methods

### Dogs

Twenty healthy client-owned adult dogs were enrolled in this prospective controlled trial. Dogs were considered eligible for this study, if they had a body weight of 10–40 kg, were older than one year and did not have any previous history of orthopedic or neurologic disorders.

Only dogs without evidence of musculoskeletal abnormalities during physical examination and upon objective gait analysis were included in this study. Dogs were excluded, if they were too large to stand completely on the balance platform in longitudinal direction. Written informed owner consent was obtained for each dog. All dogs were handled in accordance with a protocol approved by the institutional ethics committee (Nr. 258–01–03–2021, date of approval 07.06.2021). The signalment (breed, age, weight, sex, shoulder height and body condition score) of each dog was documented. Upon entry of the study, all dogs underwent complete general, orthopedic and neurologic examination. Static and dynamic balance analysis were performed at all time points, while objective gait assessments (walking and trotting) on a pressure-sensitive treadmill (CanidGait®, Zebris, Isny, Germany) were conducted only on the initial screening day and repeated at the final time point.

### Modified canine posturomed balance platform

**Fig. 2:**
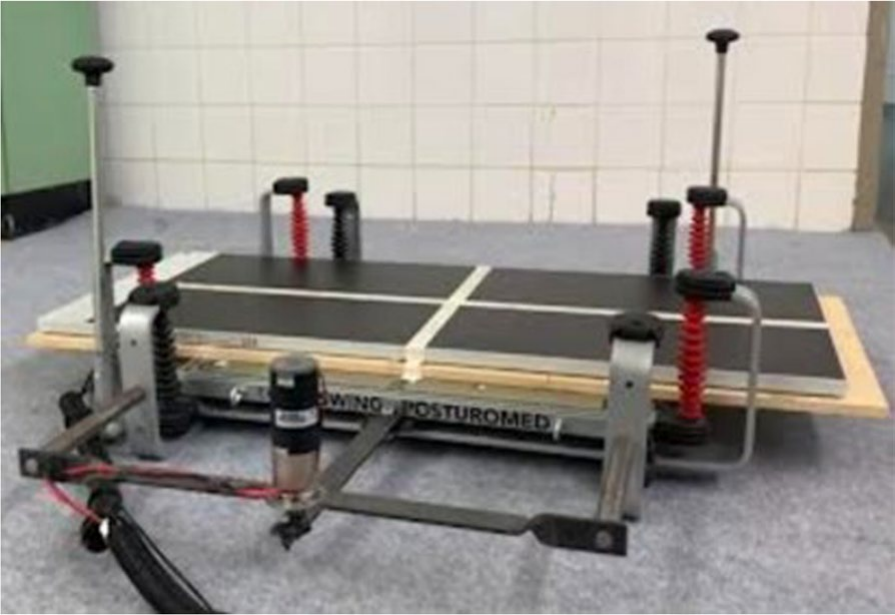
Modified canine Posturomed platform

The configuration of the Posturomed balance platform (Zebris FDM-JS System, Zebris Medical GmbH, Isny, Germany; Fig. 2), the video acquisition system (SYNCLightCam; Zebris Medical GmbH), and the associated analysis software (WinFDM Software v1.2.2; Zebris Medical GmbH; sampling rate: 100 Hz) was identical to that employed in the previous canine Posturomed study [25]. The platform utilized capacitive pressure sensors, factory-calibrated to detect pressures within a range of 1 to 80 N/cm^2^. Prior to each measurement session, the sensors were automatically zeroed during the initialization phase to eliminate baseline offset. Consequently, the platform had to remain completely unloaded during this initial step to ensure accurate calibration and prevent measurement artifacts.

### Static and dynamic balance analysis

Acclimatization—Balance analysis was conducted in a quiet room. *Acclimatization time* referred to the period required for each dog to become comfortable in the testing environment and with the presence of the platform and handlers. During this period, which lasted up to 15 min, dogs were allowed to explore the room freely and were walked on a leash three times across the platform to familiarize them with the setup before data collection began. After this acclimatization period, one test measurement was performed under static conditions with the dog standing in longitudinal direction on the unlocked platform prior to data acquisition. For balance analysis under slow-dynamic conditions, the deflection rate was set at 0.8 deflections per second (transformer frequency: 10 V) and under fast-dynamic conditions, at 1.2 deflections per second (transformer frequency: 15 V).

**Fig. 3:**
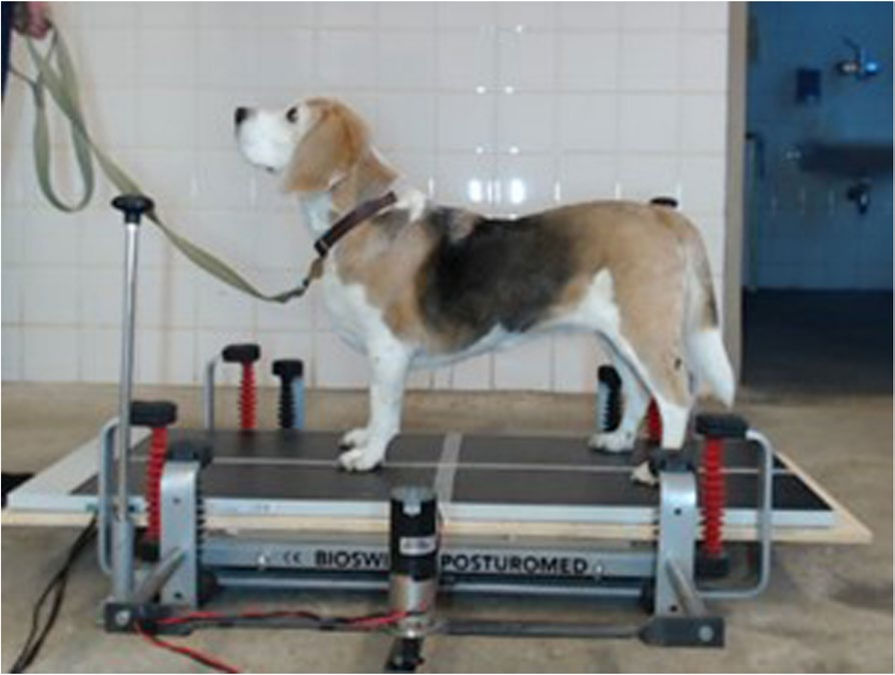
Standing Position on the modified Posturomed platform: The dogs were positioned as straight as possible longitudinally on the platform with each paw placed in its corresponding quadrant

Balance measurements – Each dog was positioned five times under static conditions, five times under slow-dynamic conditions and five times under fast-dynamic conditions longitudinally on the platform (Fig. 3). Video recordings of each trial were recorded synchronously to the COP measurements with the dogs positioned on the balance platform. Each dog was primarily handled by one single person (dog owner) for each trial at all four time points. All handlers were instructed and supported by the first author. The primary handler was standing in front of the dog and directed the dog verbally or with treats to stand as straight and symmetric as possible and without head movement on the platform. As soon as the dog remained steady and calm on the platform, a 20 s long posturographic measurement was executed for each trial. Body contact of the handler with the dog or pulling on the leash was minimized as much as possible. All dogs left the balance platform after each trial and were then repositioned on the platform for the next measurement. Measurements were continued until five valid trials per condition were obtained, without exceeding the maximum allotted evaluation time for any dog.

For trials performed under dynamic conditions, based on the individual comfort level of each dog, the transformer was gradually upregulated with the dog on the platform, until the target frequency was reached. Alternatively, the dogs were also allowed to jump directly on the already dynamized platform at target frequency. The overall evaluation time was limited to 30 min.

The same procedure was repeated three times over a period not exceeding three weeks (to allow scheduling flexibility for client-owned dogs). All follow-up assessments were conducted at times comparable to the initial evaluation to minimize potential circadian influences.

### Data analysis

Videos of each balance measurement were reviewed by one investigator (VW) to identify valid sequences for analysis. A valid 5-s sequence required that the dog stood squarely with all four limbs symmetrically positioned, maintained a steady posture without corrective stepping, and showed minimal head, body, or limb movement. Minor tail movements were accepted only if they did not affect overall postural stability. To verify this, the corresponding pressure curves were examined, and any sequences showing obvious signal changes related to tail or body motion were excluded from analysis. Five independent valid measurements for each condition were required for a successful evaluation. Each 20-s measurement represented one trial during which the dog stood on the platform under static, slow-dynamic, or fast-dynamic conditions. From each trial, one consecutive steady-state 5-s interval was analyzed, ensuring that all dogs contributed equal-length data segments under identical conditions and allowing evaluation of a stable postural phase while minimizing transient movements.

The pressure distribution of all limbs (measured in N/cm^2^ and expressed as percentage of the body weight) was measured with a sampling rate of 100 Hz. The 95% COP confidence ellipse (in mm^2^), the COP path length (in mm) and COP average velocity (in mm/sec) were calculated by the proprietary software (Animal Analysis Suite RC, Version 2.3.24). The 95% COP confidence ellipse area (CEA) was defined as the area which included 95% of all individual measured COPs within one measurement. COP path length (PL) was defined as the total length of the COP sway. COP average velocity (AV) was defined as the velocity within the COP sway [25].

### Statistical analysis

Due the presence of repeated measures generalized linear mixed effects models with individual animal as a random effect were chosen for analysis. Each valid 5-s interval was entered as a separate observation, with dog modelled as a random effect to account for repeated measurements within individuals. The following model assumptions were always checked: [1] the normality of residuals was checked by the Shapiro–Wilk normality test, [2] the homogeneity of variances between groups was checked with Bartlett test, and [3] the heteroscedasticity (constancy of error variance) was checked with Breusch–Pagan test. When assumptions were satisfied, generalized linear mixed effects models were used (R package—lmer). When assumptions were violated, robust linear mixed effects models were applied (R package—robustlmm). Both linear and robust linear models were compared using six main performance quality indicators: Akaike's Information Criterion (AIC), Bayesian Information Criterion (BIC), Conditional coefficient of determination R2, Marginal coefficient of determination R2, the intraclass-correlation coefficient (ICC) and Root Mean Square Error (RMSE). The model showing the best combination of predictive (AIC and BIC) and fitting (explanatory, R^2^, ICC, RMSE) power was preferred. All contrasts (differences) between trials or time-points were assessed after model-fitting using estimated marginal means (R package—emmeans) with Tukey adjustment for multiple comparisons. Results with a *P*-value < 0.05 were considered statistically significant. Data analysis was performed using R 4.2.1 (2022–06–23).

## Supplementary Information

Supplementary Material 1.

## Data Availability

All data supporting our outcome are included in the manuscript. If readers want additional information and/or data sets, they can be provided by the corresponding author upon reasonable request.

## Abbreviations

COP: Center of pressure
CEA: 95% COP confidence ellipse area
PL: COP path length
AV: COP average velocity
