# Supplementary material for: Learning effects during balance analysis on a modified posturomed-platform in healthy dogs
Source: BMC Vet Res. 2026 Jan 8;22:52. doi: 10.1186/s12917-025-05257-y (PMC12849616; doi:10.1186/s12917-025-05257-y)
Supplement: Supplementary file 1 — Supplementary Material 1. [file 12917_2025_5257_MOESM1_ESM.pdf]

X- and y-axis lengths of the 95% COP confidence ellipse (mean and 95% CI; mm) in 20 healthy dogs assessed at four time points under static, slow dynamic and fast dynamic conditions, including comparisons between time points

length of 95% CEA axis (X) in_mm (robust mixed-effect model)

condition = Static Condition:

timepoint	emmean	asymp.LCL	asymp.UCL
1	6.25	4.09	8.42
2	5.76	3.60	7.93
3	5.74	3.57	7.90
4	7.30	5.13	9.46

condition = Slow Dynamic Condition:

timepoint	emmean	asymp.LCL	asymp.UCL
1	28.55	26.39	30.72
2	24.91	22.74	27.07
3	24.96	22.79	27.12
4	24.55	22.39	26.71

condition = Fast Dynamic Condition:

timepoint	emmean	asymp.LCL	asymp.UCL
1	31.11	28.95	33.27
2	28.43	26.26	30.59
3	28.94	26.77	31.10
4	27.33	25.17	29.49

length of 95% CEA axis (X) in_mm (robust mixed-effect model)

condition = Static Condition:

contrast	estimate	asymp.LCL	asymp.UCL	p.value
TP1 - TP2	0.4878	-1.939	2.915	0.9552
TP1 - TP3	0.5152	-1.912	2.942	0.9478
TP1 - TP4	-1.0449	-3.472	1.382	0.6858
TP2 - TP3	0.0275	-2.400	2.455	1.0000
TP2 - TP4	-1.5327	-3.960	0.894	0.3659
TP3 - TP4	-1.5601	-3.987	0.867	0.3498

condition = Slow Dynamic Condition:

contrast	estimate	asymp.LCL	asymp.UCL	p.value
TP1 - TP2	3.6459	1.219	6.073	0.0007
TP1 - TP3	3.5964	1.169	6.024	0.0008
TP1 - TP4	4.0048	1.578	6.432	0.0001
TP2 - TP3	-0.0494	-2.477	2.378	0.9999
TP2 - TP4	0.3590	-2.068	2.786	0.9813
TP3 - TP4	0.4084	-2.019	2.836	0.9729

condition = Fast Dynamic Condition:

contrast	estimate	asymp.LCL	asymp.UCL	p.value
TP1 - TP2	2.6853	0.258	5.112	0.0232
TP1 - TP3	2.1729	-0.254	4.600	0.0980
TP1 - TP4	3.7804	1.353	6.207	0.0004
TP2 - TP3	-0.5124	-2.940	1.915	0.9486
TP2 - TP4	1.0951	-1.332	3.522	0.6527
TP3 - TP4	1.6075	-0.820	4.035	0.3228

length of 95% CEA axis (Y) in_mm (robust mixed-effect model)

condition = Static Condition:

timepoint	emmean	asymp.LCL	asymp.UCL
1	16.0	11.93	20.0
2	14.0	9.98	18.1
3	13.5	9.48	17.6
4	16.1	12.07	20.2

condition = Slow Dynamic Condition:

timepoint	emmean	asymp.LCL	asymp.UCL
1	81.3	77.28	85.4
2	70.9	66.83	74.9
3	67.4	63.36	71.4
4	62.9	58.83	66.9

condition = Fast Dynamic Condition:

timepoint	emmean	asymp.LCL	asymp.UCL
1	116.6	112.55	120.6
2	104.0	99.99	108.1
3	98.6	94.55	102.6
4	91.4	87.37	95.4

length of 95% CEA axis (Y) in_mm (robust mixed-effect model)

condition = Static Condition:

contrast	estimate	asymp.LCL	asymp.UCL	p.value
TP1-TP2	1.950	-2.258	6.16	0.6330
TP1-TP3	2.449	-1.758	6.66	0.4402
TP1-TP4	-0.143	-4.351	4.06	0.9998
TP2-TP3	0.500	-3.708	4.71	0.9901
TP2-TP4	-2.093	-6.301	2.12	0.5772
TP3-TP4	-2.593	-6.800	1.62	0.3884

condition = Slow Dynamic Condition:

contrast	estimate	asymp.LCL	asymp.UCL	p.value
TP1-TP2	10.457	6.249	14.66	<.0001
TP1-TP3	13.924	9.716	18.13	<.0001
TP1-TP4	18.455	14.248	22.66	<.0001
TP2-TP3	3.467	-0.741	7.68	0.1477
TP2-TP4	7.998	3.791	12.21	<.0001
TP3-TP4	4.531	0.323	8.74	0.0290

condition = Fast Dynamic Condition:

contrast	estimate	asymp.LCL	asymp.UCL	p.value
TP1-TP2	12.560	8.352	16.77	<.0001
TP1-TP3	18.003	13.795	22.21	<.0001
TP1-TP4	25.187	20.979	29.39	<.0001
TP2-TP3	5.443	1.235	9.65	0.0049
TP2-TP4	12.627	8.419	16.83	<.0001
TP3-TP4	7.184	2.976	11.39	0.0001
